# Glacier Calving in Greenland

**DOI:** 10.1007/s40641-017-0070-1

**Published:** 2017-10-27

**Authors:** Douglas I. Benn, Tom Cowton, Joe Todd, Adrian Luckman

**Affiliations:** 1 School of Geography and Sustainable Development, University of St Andrews, St Andrews, KY16 9AL UK; 2 Department of Geography, Swansea University, Swansea, SA2 8PP UK; 3 University Center in Svalbard, N-9171 Longyearbyen, Norway

**Keywords:** Iceberg calving, Greenland, Ice sheet models

## Abstract

In combination, the breakaway of icebergs (calving) and submarine melting at marine-terminating glaciers account for between one third and one half of the mass annually discharged from the Greenland Ice Sheet into the ocean. These ice losses are increasing due to glacier acceleration and retreat, largely in response to increased heat flux from the oceans. Behaviour of Greenland’s marine-terminating (‘tidewater’) glaciers is strongly influenced by fjord bathymetry, particularly the presence of ‘pinning points’ (narrow or shallow parts of fjords that encourage stability) and over-deepened basins (that encourage rapid retreat). Despite the importance of calving and submarine melting and significant advances in monitoring and understanding key processes, it is not yet possible to predict the tidewater glacier response to climatic and oceanic forcing with any confidence. The simple calving laws required for ice-sheet models do not adequately represent the complexity of calving processes. New detailed process models, however, are increasing our understanding of the key processes and are guiding the design of improved calving laws. There is thus some prospect of reaching the elusive goal of accurately predicting future tidewater glacier behaviour and associated rates of sea-level rise.

## Introduction

Between one third and one half of the ice lost annually from the Greenland Ice Sheet is by the breakaway of icebergs (calving) and submarine melting at the termini of marine-terminating (‘tidewater’) outlet glaciers [1]. Calving and submarine melting are collectively known as *frontal ablation* [2] and are notoriously difficult to quantify separately. Measurements from satellite or other imagery allow linear or areal frontal ablation rates to be determined (e.g. [3]), but it is rarely possible to measure the relative contribution of calving and melting below the surface. Because of these difficulties and the importance of glacier flow in governing rates of ice delivery to the termini of tidewater glaciers, frontal ablation is commonly quantified using measurements of ice discharge through a glacier cross-section or ‘flux gate’, and termed *dynamic ice loss* [4].

Submarine melting and calving processes are not independent and are not simply additive. Melting of the submerged portion of an ice front can trigger calving events, which might be several times larger than the original melted cavity [5, 6]. In addition, frontal ablation is not simply a passive process of ice removal from glacier fronts. Detachment of large masses of ice brings about geometric changes to the glacier front, which can influence glacier dynamics. In particular, loss of ice can reduce resistance to ice flow from upglacier (like removing a cork from a bottle), leading to increased discharge from the ice-sheet interior into the oceans [7, 8]. Thus, tidewater outlet glaciers can exhibit highly non-linear responses to environmental change, with major implications for rates of sea-level rise.

In recent years, there has been significant progress in understanding processes of frontal ablation and their environmental controls. Major advances have been made in observations of ice dynamics, fjord-water characteristics, processes and patterns of submarine melting, and the physics of iceberg calving [9]. The increasing richness of the observational record has been closely matched by rapid progress in numerical modelling capability [5, 10–13]. Straneo et al. [9] highlighted three priorities for future research: (1) process studies targeting specific dynamic regimes, (2) sustained observations of key localities, and (3) synthesis of the results into improved process models and parameterizations. Major challenges remain in all three areas before the glaciological community can achieve the important goal of predicting the dynamic response of the Greenland Ice Sheet to climatic and oceanic forcing. In this paper, we review recent work on tidewater glaciers in Greenland, including their overall contribution to mass loss, ice-front variations, frontal ablation processes, and modelling efforts. We conclude by identifying important new directions in tidewater glacier research and remaining challenges for the future.

## Contribution of Calving Glaciers to Greenland’s Mass Budget

**Fig. 1:**
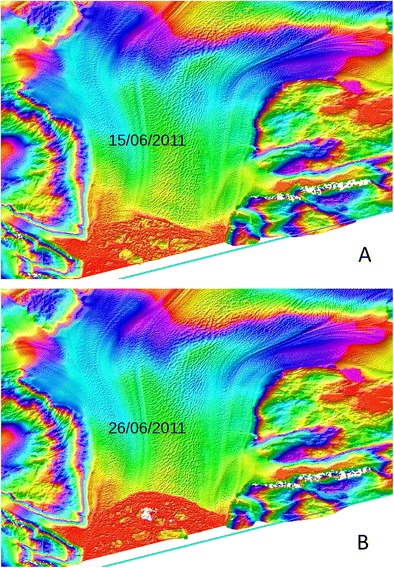
The terminus of Helheim Glacier before **(a)** and after (**b**) large calving events in June 2011. The images are derived from TanDEM-X data, processed by Suzanne Bevan

Dynamic ice loss is a major factor in the mass balance of the Greenland Ice Sheet. In 2000, the total discharge from all of Greenland’s tidewater outlet glaciers was 462 ± 6 Gt [1]. Fifteen glaciers make up 50% of the total, and only five (Jakobshavns Isbrae, Kangerdlugssuaq, Koge Bugt, Ikertivaq South, and Helheim) account for > 30% (Fig. 1). Total discharge rate increased by 18% to 546 ± 11 Gt/year between 2000 and 2012, but inter-annual variability is high, particularly in SE Greenland [1]. Dynamic ice losses were roughly equal to surface melt and runoff in the period 2000–2008, but the share was somewhat less during the exceptionally high-surface melt years of 2009–2012.

Taken together, dynamic ice losses and runoff of surface meltwater exceed snow accumulation over the Greenland Ice Sheet, so the overall mass balance of the ice sheet is negative. Analysis of surface elevation and mass changes from CryoSat-2 and GRACE satellite data indicates that the Greenland Ice Sheet had a net annual mass balance of − 269 ± 51 Gt/year from Jan 2011–Dec 2014 [14]. Dynamic ice loss from marine outlet glaciers makes a large contribution to this deficit, particularly from Kangerdlugssuaq in the east, Jakobshavns Isbrae, Upernavik Isstrøm and Steenstrup Glacier on the west coast, and Zachariæ Isstrøm in the north-east.

It has only recently become possible to quantify the dynamic contribution to mass loss from the whole Greenland Ice Sheet, and it is unwise to infer long-term trends from short-term data. However, long-term records of iceberg losses can be determined from monthly iceberg counts by the US Coastguard’s International Ice Patrol, which record all bergs greater than 5 m in above-water length south of a line extending along 48° N from the Newfoundland coast to approximately 40° W. The data (beginning in 1900) display large inter-annual variability, but clearly show much higher overall iceberg frequencies since the late twentieth century than in earlier periods [15, 16].

## Ice-Front Variations of Calving Glaciers

**Fig. 2:**
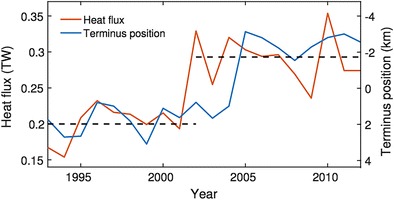
Modelled annual mean up-fjord heat flux (red) and observed terminus retreat (blue) at Kangerdlugssuaq Glacier, east Greenland. Variability in heat flux reflects variability in both ice-sheet runoff (which drives fjord circulation) and shelf water temperature. Black dashed lines show mean values for the periods 1993–2001 and 2002–2011. Glacier terminus positions are based on processing of remote sensing imagery by Seale et al. [23] and Bevan et al. [17], supplemented with additional data from digitisation of Landsat scenes. Terminus positions are shown as anomalies with respect to the mean over the time series, with a decrease indicating glacier retreat (note the inverted scale on the vertical axis). Modified from Cowton et al. [20]

Increasing dynamic ice loss from the Greenland Ice Sheet reflects a combination of increased ice velocities and ice-front retreat [4, 17–19]. Correlations between ice-front position change, and air and sea temperatures suggest that tidewater glaciers are sensitive to both atmospheric and oceanic forcing ([20–22]; Fig. 2), although the physical processes underlying such correlations are not fully understood. Ocean forcing is particularly important in SW and SE Greenland, where incursions of warm water into fjords have been associated with episodes of glacier acceleration and retreat [18]. For example, rapid retreat of Kangerdlugssuaq Glacier coincided with a 50% increase in ocean heat available for melting at the ice front ([20]; Fig. 2).

The overall trend of glacier acceleration and retreat masks considerable regional variation [24]. Greenland’s tidewater outlet glaciers commonly exhibit asynchronous ice-front behaviour, and some glaciers may remain stationary or advance while others are in retreat. Indeed, this is true even for glaciers terminating in the same fjord system [25, 26]. This asynchronous behaviour may partly reflect local variations in ocean heat flux and other forcing, but local fjord and glacier bed bathymetry clearly play an important role through their influence on the mechanical stability of ice fronts. The behaviour of tidewater glaciers is strongly dependent on the presence of shallow sills or narrow sections of a fjord (‘pinning points’) where calving is suppressed [11, 27]. Major pinning points may allow glaciers to maintain stable terminus positions for many decades, while other adjacent glaciers undergo retreat (e.g. Store Glacier: [28–30]). Conversely, detachment from a pinning point may initiate episodes of rapid thinning and retreat, especially where fjords deepen inland [31]. Recent asynchronous behaviour of Zachariae Isstrom and Nioghalvfjerdsfjord Glacier is largely attributable to variations in fjord bathymetry, with rapid retreat of the former following its detachment from a shallow sill into deepening water while the latter is retreating slowly along an upward-sloping bed [32]. The increasing availability of bed data now makes it possible to identify vulnerable glaciers from the presence of over-deepened basins beneath their tongues [33].

## Processes of Frontal Ablation

Greenland tidewater glaciers have variable characteristics, with important implications for processes of frontal ablation. Some glacier termini are fully buoyant forming *floating ice tongues*, while others rest on the sea floor forming *grounded ice fronts*. Both of these conditions may occur on different parts of the same glacier front, while other glaciers may undergo temporal transitions between grounded and buoyant states. Research into processes of frontal ablation in Greenland has focused on two main mechanisms: *submarine melting of ice fronts* (and associated calving) and *buoyancy-driven calving*.

Observations have revealed the presence of warm subsurface waters of Atlantic origin in the upper reaches of Greenland’s deep fjords [9, 34, 35], raising interest in the role of submarine melting at tidewater outlet glaciers [36]. Submarine melting is a direct source of mass loss from tidewater glaciers, but it may also serve to accelerate calving by undercutting grounded glacier termini [2, 3, 6] or weakening floating ice tongues [37]. Establishing the impact and significance of submarine melting at Greenland’s tidewater glaciers has proven challenging, however, due to the extreme difficulty of data collection. Estimates of submarine melt rate have been derived primarily from fjord flux gate studies, involving the analysis of heat and salt budgets across hydrographic sections that may lie several kilometres down-fjord from the calving front [38–41]. These studies indicate that average calving front melt rates lie in the range of ~ 1–10 m/day, with the spread of values reflecting regional differences in ocean heat content and subglacial discharge, in addition to the uncertainties introduced by temporal variability in fjord circulation, incomplete sampling across fjord sections, and the input of freshwater from iceberg melt [42, 43]. Furthermore, whole-fjord estimates made over short periods of time may mask considerable spatial and temporal variability, making the evaluation of the likely effects on calving processes difficult.

Recently, new insights into spatial variability in submarine melting have emerged from sonar imaging of several glacier calving fronts in West Greenland [38, 44, 45]. The surveys indicate widespread undercutting, with localized zones undercut by as much as one ice thickness. These zones are thought to correspond to locations at which glacial meltwater is discharged subglacially across the grounding line, rising buoyantly and mixing with the relatively warm fjord waters to form turbid plumes [46] that can be observed both visually and in hydrographic data (e.g. [47]). Modelling studies indicate that submarine melt rate increases linearly with water temperature and with the subglacial discharge of meltwater runoff raised to a power between one-third and three-fourths [48–50], supporting the interpretation of these highly undercut zones as areas of enhanced plume-driven melting.

These findings indicate that melt-undercutting is sensitive to ocean temperature, glacier runoff, and the morphology of the subglacial hydrological system. Runoff input from a single channel produces a small zone of highly concentrated melting, whereas distributing runoff more evenly across the width of the glacier decreases the maximum local melt rates but can increase the total submarine melting of the calving front by a factor of 5 [51, 52]. This is likely a significant consideration with respect to the effect of melt-undercutting on calving: small melt rates distributed across the full terminus width may have a different impact on terminus stability than one or a few zones of focused melting (see below). The subglacial hydrology of tidewater glaciers remains poorly understood, but terminus morphology [38, 44, 45] and plume properties [53–55] indicate that it may lie typically between the channelized and distributed end members, with a substantial proportion of meltwater input from several broad (> 100-m diameter) subglacial channels.

While it is clear that melting below the waterline preconditions calving events, the precise relationship between melt-undercutting and calving remains unclear. Undercutting of grounded glacier fronts removes support from the overlying ice, potentially triggering calving events that may be smaller or larger than the submarine cavity. If calving events are consistently smaller than the cavity, long-term frontal ablation rates will simply be determined by the submarine melt rate. On the other hand, stress migration effects may trigger calving events larger than the submarine cavity, in which case long-term frontal ablation rates will be greater than the submarine melt rate [5, 6]. In addition, localized melt-undercutting may encourage calving of other parts of the glacier front. For example, at Store Glacier, Chauché et al. [47] observed the formation of embayments in the ice front in the vicinity of upwelling meltwater plumes. Growth of embayments removes lateral support from intervening headlands, reducing their mechanical stability and encouraging calving.

The impact of submarine melting also depends on glaciological factors such as the degree of buoyancy and rates of ice flow. In general, the impact of melt-undercutting should be greatest on well-grounded glaciers, because glacier fronts close to buoyancy are largely supported by water pressure and should be less affected by the loss of ice at the base. However, submarine melting is clearly implicated in calving and break-up of floating glacier tongues in Greenland. For example, the loss of the 15 km floating ice tongue of Jakobshavn Isbrae after 2001 was preceded by a 1 °C increase in ocean temperatures, enough to increase submarine melting by 25% [2, 56, 57]. At Petermann Glacier, North Greenland, major calving events occurred in 2010 and 2012, releasing tabular icebergs ~ 250 and 130 km^2^ in area, respectively. In the years preceding the first event, the floating ice tongue thinned by 5 m/year, largely because of basal melting [37]. This thinning appears to have played a key role in structural weakening, possibly exacerbated by the presence of channels incised into the base of the ice tongue. The wider significance of these events is difficult to assess, and Falkner et al. [58] noted that a massive ice-retreat event of a similar magnitude to that on Petermann Glacier in 2010 might have occurred during a gap in the observational record. However, there is no direct evidence that this was the case, and following another high-magnitude calving event in 2012, the glacier was about 25 km more retreated than any observed position since records began in 1876 [37].

If other factors are equal, melt-undercutting will likely have a larger impact on glacier evolution if submarine melt rates exceed rates of ice flow. However, even fast-flowing glaciers can be significantly impacted by submarine melting if, for example, loss of a floating ice tongue forces the glacier into a different dynamical regime [2].

**Fig. 3:**
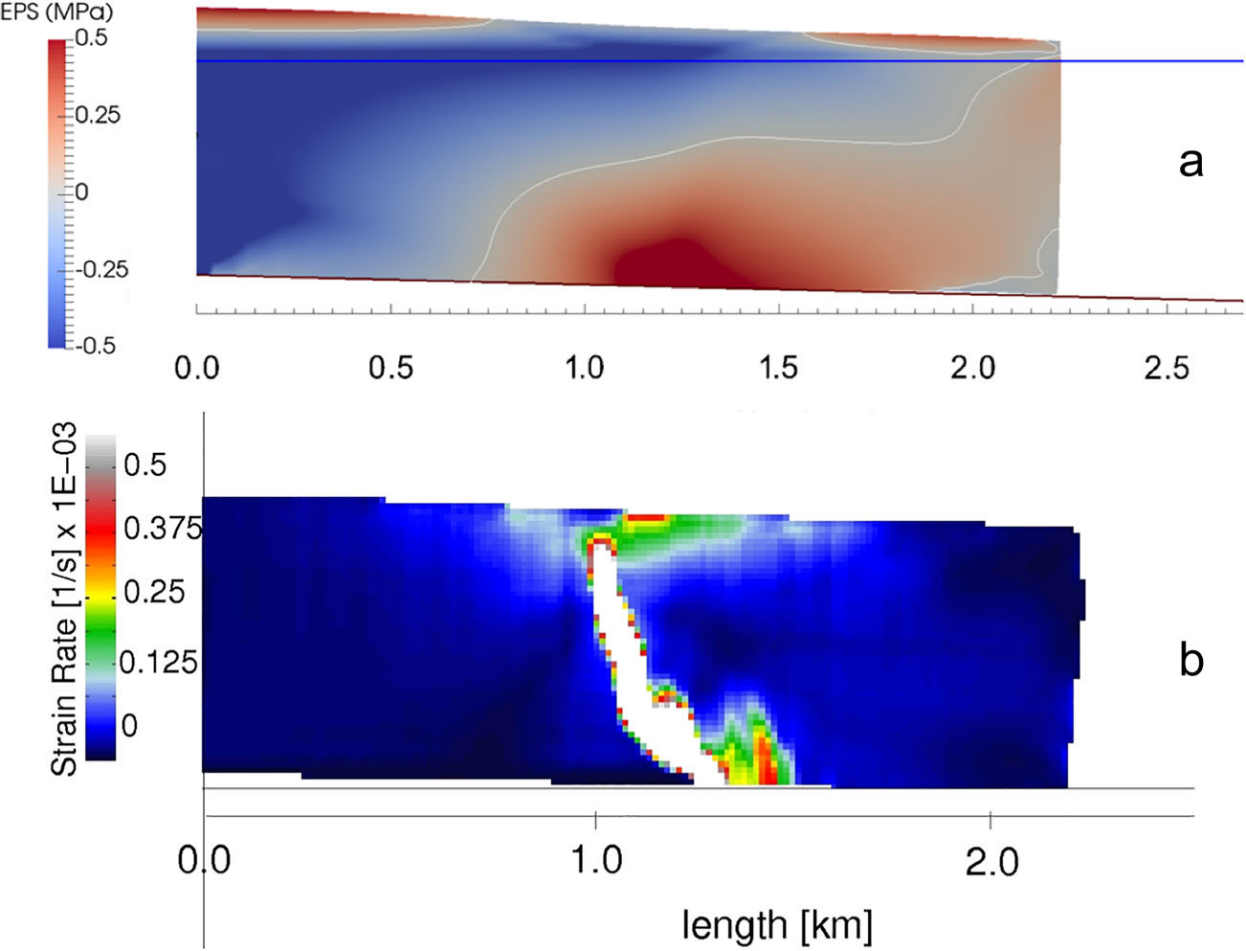
Simulations of buoyancy-driven calving. **a** Stress field in a super-buoyant ice tongue modelled in Elmer/Ice. EPS is the effective principal stress, defined as the principal component of the Cauchy stress tensor plus subglacial water pressure. **b** Strain rates in inter-particle bonds during a simulation with the Helsinki Discrete Element Model (HiDEM), using the same geometry as in **a**, with broken bonds shown in white. Values are averages across the model domain, giving the misleading impression of a wide fracture zone. This snapshot shows the propagation of narrow fractures upward from the centres of the concentration in tensile stress at the base. Modified from Benn et al. [5]

*Buoyancy-driven calving* is an important process on large, fast-flowing outlet glaciers [59–64]. Rapid ice flow into deep water can create ‘super-buoyant’ conditions, in which ice fronts are out of hydrostatic equilibrium and subject to large upward-directed torque forces (Fig. 3). This results in rotation and uplift of the glacier tongue around a ‘flexion zone’ located near the ungrounding point [59, 61]. Geometric considerations show that block rotation is associated with the growth of basal crevasses, which eventually lead to calving and overturning of the terminal block, usually closely followed by block disintegration. Buoyancy-driven calving events can be very large, typically affecting the full thickness of the glacier for many hundreds of metres across- and along-flow [59, 60, 64]. Collisions between capsizing bergs and the glacier terminus are the likely source of seismic signals associated with large calving events [65–67]. Detailed observations by Murray et al. [68] show that seismic energy release is associated with distinctive cycles of elastic frontal compression and re-extension of the remaining glacier front in response to the release and capsize of large bergs.

Both buoyancy-driven calving and melt-undercutting can occur on different parts of the same glacier front. Analysis of Extreme Ice Survey time-lapse camera data on Rink Glacier by Medrzycka et al. [60] has shown that frequent, small calving events occur in the vicinity of upwelling plumes, whereas infrequent, large, buoyancy-driven events occur where the glacier terminates in deep water. It is likely that the relative contribution of buoyancy-driven and melt-undercutting (and other calving processes such as the release of tabular bergs in response to longitudinal extension) varies greatly between glaciers in Greenland. As yet, however, observations are too few to provide a reliable overview.

Ice-front behaviour is also strongly influenced by the presence of mélange, a mixture of calved icebergs and sea ice. In winter, when sea ice is well frozen, mélange forms a strong, rigid mass, whereas in summer, calved bergs have greater freedom to move relative to one another. Howat et al. [69] and Moon et al. [24] found that a reduction in calving losses in NW Greenland commonly coincides with the formation of rigid iceberg mélange, whereas summer ice-front retreat is associated with mélange break-up. Rigid mélange can suppress calving and iceberg overturn by applying back-pressure on the glacier front [66, 67, 70–72]. This effect is especially strong where fjord topography encourages ‘dynamic jamming’ [73].

## Modelling

To predict the future evolution of the Greenland Ice Sheet, it is necessary to include ‘calving laws’ in numerical ice-sheet models. However, simulating the evolution of the whole ice sheet over many time-steps places huge demands on computer resources, so it is necessary to simplify calculations by adopting approximations of the underlying physics. In turn, this means that calving laws also need to be simple and easy to implement. The calving laws currently in use either predict calving *location* based on theoretical crevasse penetration depths or yield criteria [74–78] or predict calving *rates* from empirical functions (e.g. [28, 29]). Nick et al. [79] used a crevasse-depth calving law implemented in a vertically integrated flowline model to predict future ice losses and sea-level contribution from four major outlet glaciers in Greenland. Similar models were used by Lea et al. [80] to simulate the historical behaviour of Kangiata Nunaata Sermia (KNS) and by Enderlin et al. [27] to explore topographic controls on glacier behaviour using synthetic geometries representative of Greenland glaciers. In contrast, Ultee and Bassis [13, 78] used physical considerations to define simple calving criteria that have proved very effective at reproducing observed patterns of glacier terminus advance and retreat, including Jakobshavn Isbrae and Helheim Glacier.

While they can replicate the general behaviour of tidewater glaciers (e.g. rapid retreat into over-deepened basins), vertically integrated models struggle to capture the effects of processes that may be key controls of tidewater glacier behaviour. For example, the effects of melt-undercutting on frontal ablation cannot be represented explicitly in vertically integrated models and must be parameterized by applying uniform melt rates to the glacier front (e.g. [28, 29, 79]). Similarly, buoyant calving processes cannot be represented in vertically integrated models, because they assume buoyant equilibrium at the glacier front and omit the key upward-directed forces that trigger calving.

These problems have been overcome by the development of efficient calving routines in the ‘full-Stokes’ finite element model Elmer/Ice in both two and three dimensions [30, 77]. The 3-D implementation in particular is a major step forward in calving model capability and includes a crevasse-depth calving law generalized to include all stresses and parameterizations of both distributed and concentrated meltwater plumes and the effects of ice mélange. When applied to Store Glacier, the model impressively replicates seasonal fluctuations of the glacier front without the need for model tuning [30]. Fidelity to observed processes includes the prediction of large calving events in super-buoyant regions and calving of promontories created by localized plume meltings melting.

Another major breakthrough is the application of *discrete element models* to modelling frontal ablation processes [81–83]. Discrete element models represent ice as assemblages of particles connected by breakable bonds and can thus explicitly simulate fracture and calving processes. Systematic experiments with the Helsinki Discrete Element Model (HiDEM) show that the full range of observed calving processes emerges spontaneously in response to changing boundary conditions (buoyancy, undercutting, gradients in basal friction), allowing detailed analysis of calving processes and their controls [5]. Such models are computationally expensive, limiting their application to single calving events. However, routines have been developed to allow exchange of geometries between HiDEM and Elmer/Ice, so that the former can be used to simulate individual calving events and the latter used to evolve ice geometry between events [5, 84]. By switching back and forth between HiDEM and Elmer/Ice, the evolution of tidewater glaciers can be simulated in unprecedented detail (Fig. 3).

These high-resolution models are not currently practical at the ice-sheet scale or for long-term simulations, due to their very high computational cost. However, they offer valuable insights into frontal ablation processes, which can guide the development of the simple calving parameterizations required for ice-sheet models [5]. Experiments with high-fidelity process models, combined with targeted observations, may yet achieve the elusive goal of defining robust physically based calving laws applicable at the ice-sheet scale.

## Conclusions and Priorities for Future Research

In the last few years, significant advances have been made in understanding frontal ablation of Greenland tidewater glaciers, reflecting both theoretical advances and intensive development of new monitoring and modelling techniques (e.g. [30, 59, 61, 68, 81, 85]). Exciting new opportunities have been created by the advent of remotely controlled or autonomous vehicles [86, 87]. Additional focused effort is required to achieve the important goal of predicting dynamic ice losses from the whole Greenland Ice Sheet, and hence their future contribution to sea-level rise. We identify three research priorities, which parallel those highlighted by Straneo et al. [9]: (1) filling remaining gaps in our understanding of frontal ablation processes, (2) obtaining high-resolution data for model initialization and validation, and (3) the development of both detailed process models and simple but robust calving laws.

The main frontal ablation processes are now reasonably well known, at least in outline, but continued research effort will be required to obtain a comprehensive, fully quantitative understanding of the key processes and their controls. In particular, concurrent data on fjord circulation and heat flux, glacier hydrology and plume dynamics, mélange characteristics, melt-undercutting, and calving are needed to establish the links between ocean forcing and glacier response. The difficulty of obtaining representative data for model initialization and validation is exacerbated by the high spatial and temporal variability of both glaciological and oceanographic conditions around Greenland. Thanks to major projects such as Operation Icebridge, Oceans Melting Greenland (OMG), and other programs (e.g. [28, 29, 88]), a wide range of high-resolution data is becoming available. Improvements in modelling techniques, however, bring increased data requirements. Due to the strong influence of topography on tidewater glacier behaviour, glacier bed elevation and fjord bathymetry data are essential for successful modelling. Bed data are now available for many key areas through a combination of airborne ice-penetrating radar and gravimetric surveys and numerical inversion of surface velocity data (e.g. [89, 90]). Similarly, direct measurements of submerged ice fronts and oceanographic data from ice-proximal fjord environments are needed for ‘ground truthing’ models of melt-undercutting.

In terms of numerical modelling, the current research effort is focused on two complementary problems: (1) the refinement and application of detailed process models [5] and (2) the development of the simple parameterizations required for ice-sheet models (e.g. [78]). The advent of discrete element models such as HiDEM [81] and efficient calving routines in Elmer/Ice [30] has opened up the possibility of simulating the full range of frontal ablation processes in unprecedented detail. An important goal for the future is to couple HiDEM and Elmer/Ice with oceanographic models, to allow simulation of submarine melt and calving processes and their interactions. To date, models of frontal ablation have focused on one aspect of the system, either using plume models to simulate changes in ice-front geometry (e.g. [50, 54]) or using specified undercut geometries to explore the impact on calving (e.g. [5, 6]). By coupling plume and calving models, it will become possible to explore feedbacks between undercutting and calving and to predict the response of tidewater glaciers to changes in fjord temperatures, meltwater discharge, and other factors.

High-fidelity process models are very different in conception from the simple calving laws needed for ice-sheet models. Convergence of these two contrasting approaches, however, offers the prospect of reaching the elusive goal of accurately predicting future tidewater glacier behaviour and associated rates of sea-level rise.
